# The Effect of Combined Exposure of 900 MHz Radiofrequency Fields and Doxorubicin in HL-60 Cells

**DOI:** 10.1371/journal.pone.0046102

**Published:** 2012-09-28

**Authors:** Zongda Jin, Chunyan Zong, Bingcheng Jiang, Zhen Zhou, Jian Tong, Yi Cao

**Affiliations:** School of Public Health, Soochow University, Suzhou, Jiangsu, People's Republic of China; University of Manitoba, Canada

## Abstract

Human promyelocytic leukemia HL-60 cells were pre-exposed to non-ionizing 900 MHz radiofrequency fields (RF) at 12 µW/cm^2^ power density for 1 hour/day for 3 days and then treated with a chemotherapeutic drug, doxorubicin (DOX, 0.125 mg/L). Several end-points related to toxicity, viz., viability, apoptosis, mitochondrial membrane potential (MMP), intracellular free calcium (Ca^2+^) and Ca^2+^-Mg^2+^ -ATPase activity were measured. The results obtained in un-exposed and sham-exposed control cells were compared with those exposed to RF alone, DOX alone and RF+DOX. The results indicated no significant differences between un-exposed, sham-exposed control cells and those exposed to RF alone while treatment with DOX alone showed a significant decrease in viability, increased apoptosis, decreased MMP, increased Ca^2+^ and decreased Ca^2+^-Mg^2+-^ATPase activity. When the latter results were compared with cells exposed RF+DOX, the data showed increased cell proliferation, decreased apoptosis, increased MMP, decreased Ca^2+^ and increased Ca^2+^-Mg^2+^-ATPase activity. Thus, RF pre-exposure appear to protect the HL-60 cells from the toxic effects of subsequent treatment with DOX. These observations were similar to our earlier data which suggested that pre-exposure of mice to 900 MHz RF at 120 µW/cm^2^ power density for 1 hours/day for 14 days had a protective effect in hematopoietic tissue damage induced by subsequent gamma-irradiation.

## Introduction

Cancer is a major cause for human suffering in the world with an average of 10 million new cases diagnosed every year [1], [2]. Chemotherapy and/or radiotherapy are effective in the treatment of a wide range of malignancies. However, these therapies involve repeated exposures that are toxic not only to cancer cells but also to healthy normal cells. Myelosuppression is the most common side effect resulting in anemia, thrombocytopenia and febrile neutropenia which often lead to increased susceptibility to many types of infections and can be fatal in a small proportion of patients [3], [4]. Doxorubicin (DOX or adriamycin) is the most commonly used anticancer drug because of its efficacy against various tumors. Like many other chemotherapeutic drugs, administration of DOX has toxic effects on hematopoietic cells [5], [6].

Radiofrequency fields (RF) in the frequency range 300 MHz to 300 GHz have a significant and positive impact in modern society. They are increasingly used in wireless communication systems, radar and space research, industrial processing and medicine. The observations published in recent reports have indicated that animals and human cells which were pre-exposed to RF were resistant to harmful effects from subsequent γ-irradiation and a chemotherapeutic drug, respectively [7]–[9]. The results from our own earlier studies have also indicated that pre-exposure of the mice to 900 MHz at 120 µW/cm^2^ power density for 14 days had ‘protective’ effect on hematopoietic tissue following γ-irradiation [10], [11]. The aim of this investigation was to examine whether a similar protective response would be elicited when human promyelocytic leukemia HL-60 cells (derived from hematopoietic tissue) were pre-exposed to 900 MHz at 12 µW/cm^2^ power density for 3 days and then treated with a chemotherapeutic drug, doxorubicin (DOX).

## Materials and Methods

### Cells and Reagents

HL-60 cells were obtained from the Toxicology department of Public Health School of Soochow University, Suzhou city, China (original source - American Type Culture Collection, VA, USA. ATCC# CCL-240). RPMI 1640 medium, fetal bovine serum (FBS) and phosphate buffered saline (PBS) were purchased from Gbico, Shanghai, China. N-2-Hydroxyethylpiperazine-N′-2-ethanesulfonic acid (HEPES), L-glutamine, penicillin and streptomycin were obtained from Bio Basic Inc., Shanghai, China. Annexin V-FICT/PI was purchased from KeyGEN Biotech. Co., Ltd., Suzhou, China. JC-1 and Fluo-3-AM were from Molecular Probes, Suzhou, China. Ca^2+^-Mg^2+^-ATPase commercial kit was obtained from Nanjing Jiancheng Bioengineering Institute, Nanjing, China.

### HL-60 Cell Cultures

HL-60 cells in passages 3–5 were grown in suspension cultures in RPMI 1640 medium supplemented with 2 mM L-glutamine, 2.5 mM HEPES, 100 U/ml penicillin, 100 µg/ml streptomycin and 10% heat-inactivated FBS (vol/vol). Cultures were maintained in an incubator at 37+/0.5°C with humidified atmosphere of 5% carbon dioxide and 95% air. Sub-culturing was done every 2–3 days when the cell density reached >10^6^ cells/ml.

### RF Exposure

**Figure 1:**
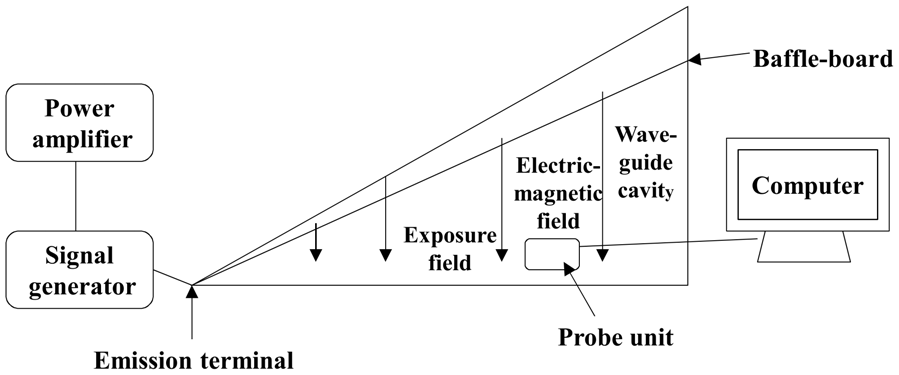
Schematic diagram of 900 MHz RF exposure device.

The RF exposure system was built in-house and consisted of a GTEM cell (Gigahertz Transverse Electromagnetic Cell, Soochow University, Suzhou, China), signal generator (SN2130J6030, PMM, Cisano sul Neva, Italy) and a power amplifier (SN1020, HD communication Corp. Ronkonkoma, NY, USA). The continuous wave 900 MHz RF signal emitted by the generator was magnified initially and then fed into GTEM cell through an antenna (Southeast University, Nanjing, Jiangsu, China). The RF field inside the GTEM was probed using a field strength meter (PMM, Cisano sul Neva, Italy) and the precise positions which provided power densities of 12, 120 or 1,200 µW/cm^2^ were determined. The required power density (12 µW/cm^2^ to assess the RF+/−DOX effect) was continuously monitored and recorded every 5 minutes using a computer controlled data logging system which indicated 12.178+/−0.003 µW/cm^2^ during the 1 hour RF exposure (Figure 1) [11]. Four petri-dishes each containing 5×10^5^ cells/ml (4 ml medium total) were placed on a non-conductive table/platform at a height of 100 cm at the precise location where the required power density was measured. The distance between the petri-dishes and the exposure unit (probe) was 18 cm while the distance between two petri-dishes was 5 mm. The height of the medium when the cells were settled as monolayer at the bottom in each petri-dish (before RF exposure began) was 1.5 mm. The direction of propagation of the incident field was parallel to the plane of the medium. The SAR at the input 12 µW/cm^2^ power flux density of was calculated using Burkhardt's formula [12]. At the bottom of the petri-dish, the peak and average SARs estimated for 900 MHz exposure at 12 µW/cm^2^ power density was extremely low, 4.1×10^−5^ and 2.5×10^−5^ W/kg, respectively. The GTEM was installed in a room which maintained 37+/−0.5°C temperature (relative humidity of 87%, without CO_2_) and the inside temperature of GTEM was also the same.

### Doxorubicin

Doxorubicin hydrochloride (purity >98%, Shenzhen Main Luck Pharmaceuticals Inc., Suzhou, China) was prepared in RMPI 1640 serum-free medium at a concentration of 10 mg/L and stored at −20°C as stock solution. Further dilution, 0.125 mg/L, was made in the same medium just before treating the cells.

### RF and/or DOX exposure

Cells in log-phase of growth were used for all experiments which were repeated twice. Separate cultures (5×10^5^ cells/ml) were set up in 60 mm petri-dishes, each using 4 ml complete medium for the following treatments: (i) un-exposed controls, (ii) sham-exposed controls, (iii) RF alone, (iv) DOX alone, and (v) RF+DOX. The RF exposure was for 1 hour/day for 3 days and the exposure time each day was between 9 AM and 10 AM. Un-exposed controls were placed in an incubator (37+/−0.5°C) in the laboratory. Sham-exposures were conducted in the same GTEM system without RF transmission. Cells which were designated for DOX alone and RF+DOX were treated with DOX, 0.125 mg/L, for 48 hours, on the day after the last RF exposure, i.e., on the 4th day.

### Viability

A preliminary experiment was conducted to determine the minimum power density needed for 900 MHz RF pre-exposure to minimize the damage induced by subsequent exposure to DOX. Cells were pre-exposed to 900 MHz RF at 12, 120 and 1200 µW/cm^2^ power density for 1 hour/day for 3 days. Then, 3×10^4^ cells were seeded in each well in a 96-well plate and cultured in the presence of DOX for 48 hours. The viability of the cells was assessed using the CCK-8 assay which is based on the ability of viable cells to convert the 2-(2-methoxy-4-nitrophenyl)-3-(4-nitrophenyl)-5-(2, 4-disulfophenyl)-2H-tetrazolium, monosodium salt into a water-insoluble, orange-colored formazan. Then, CCK-8 was added to each well and the cells were incubated at 37°C for 3 hours. Then, the absorbance in each well was determined using a Microplate Reader (Bio Tek) with the test wavelength of 450 nm and the reference wavelength of 650 nm. The viability of the cells was found to be maximal when the cells were exposed to 900 MHz at 12 µW/cm^2^ +DOX and then decreased with increased power-density. Hence, the 900 MHz RF exposure at 12 µW/cm^2^ +/− DOX treatment was used for the following experiments.

### Apoptosis

Un-exposed, sham-exposed, RF alone, DOX alone and RF+DOX exposed cells were collected, washed twice with ice-cold PBS and centrifuged at 100×g for 5 min. The cell pellet was re-suspended in 400 µl binding buffer and stained with commercially available kit containing annexin V-FITC and propidium iodide (5 µl each) for 15 min at 37°C followed by immediate analysis using flow cytometer (FC 500, Becton Dickinson). The early stage apoptotic cells were distinguished by positive green staining with Annexin V-FITC while dead cells are stained red with propidium iodide.

### Mitochondrial Membrane Potential (MMP)

MMP was measured using JC-1 (5, 5′, 6, 6′-Tetrachloro-1, 1′, 3, 3′-tetraethyl benzimidazolyl carbocyanine iodide) which is the most reliable probe for assessing changes in MMP in intact and live cells [13]. JC-1 stains the mitochondria in cells with high membrane potentials by forming J-aggregates that emit orange-red fluorescence (FL-2) at 590 nm upon excitation at 488 nm. In cells with depolarized or damaged mitochondrial membrane, JC-1 displays green fluorescence (FL-1) at 525 nm with the same excitation wavelength. Un-exposed, sham-exposed, RF alone, DOX alone and RF+DOX exposed cells were collected, washed twice with PBS and 400 µl of JC-1 (final concentration is 10 µg/ml) was gently added to the cells and incubated for 30 minutes at 37°C in the dark. Then, the cells were washed with PBS and analyzed immediately using flow cytometer (FACScan; BD Biosciences). In each sample, a total of 10,000 cells were analyzed for orange-red fluorescence with a 590 nm filter and green fluorescence with a 525 nm filter. A higher red: green ratio indicates hyperpolarized mitochondrial inner membrane.

### Intracellular Ca^2+^ Levels

Un-exposed, sham-exposed, RF alone, DOX alone and RF+DOX exposed cells were collected, washed twice in PBS, loaded with 400 µl of Fluo3-AM (10 µmol/ml) and incubated for 30 minutes in the dark. In each sample, the fluorescence intensity was measured in 10,000 cells, using flow cytometry, at an excitation wavelength of 488 nm and an emission wavelength of 526 nm. The intensity of the fluorescence depends on the concentration of free calcium, i.e., the greater fluorescence intensity, the higher the free calcium concentration.

### Ca^2+^-Mg^2+^-ATPase Activity

Un-exposed, sham-exposed, RF alone, DOX alone and RF+DOX exposed cells were collected, washed twice in PBS and the activity of Ca^2+^-Mg^2+^-ATPase was determined using a commercial kit. In each sample, the inorganic phosphate (Pi) liberated from ATP hydrolysis was measured using 756 UV-Vis Spectrophotometer (Shanghai Spectrum Instrument Co., Ltd., Shanghai, China). The DOX treatment for this test was only 24 hours.

### Statistical Analysis

All data were subjected to statistical analysis. One-way analysis of variance (ANOVA) and, Least Significant Difference (LSD) test was used when appropriate. Pearson's correlation coefficient analysis was performed using Student's *t* test. Linear regression between % apoptosis and intracellular free Ca^2+^ was calculated. A *p* value<0.05 was used for significant differences between exposures. The results presented are mean +/− standard deviations (SD).

## Results

**Figure 2:**
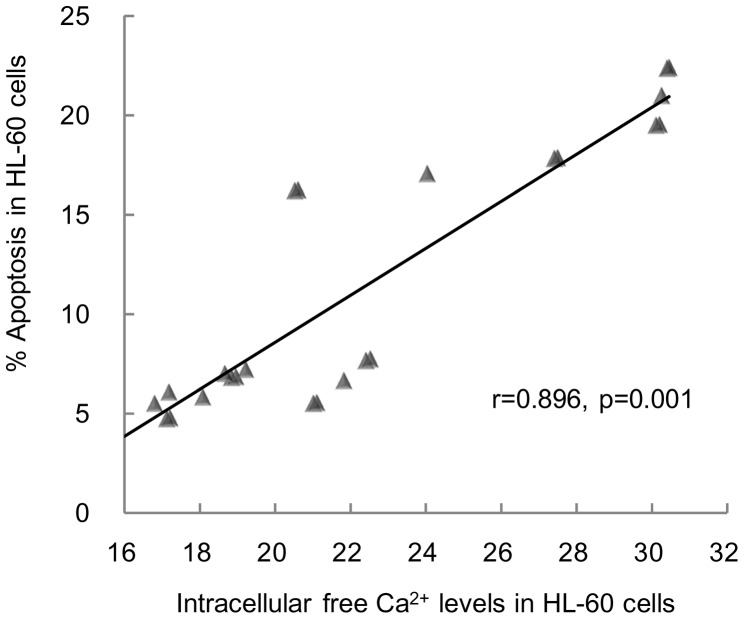
Correlation between % apoptosis and intracellular free Ca^2+^ levels in HL-60 cells.

All data are presented in Table 1. The results obtained in the preliminary experiment indicated that the viability of cells decreased with increased power density used: 83%, 61% and 59% viable cells when exposed to RF at 12, 120 and 1200 µW/cm^2^ power density, respectively. The observations made in un-exposed and sham-exposed cells were identical: 100% viability, 6% apoptosis, fluorescence intensity (FI) of 87 for MMP, FI of 18 for intracellular free Ca^2+^ and 17 U/mgprot for Ca^2+^-Mg^2+^-ATPase activity. There was a significant and positive correlation between % apoptosis and intracellular free Ca^2+^ (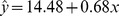) (Figure 2). The results in cells exposed to RF alone at 12 µW/cm^2^ were also similar to those in un-exposed and sham-exposed cells: 99% viability, 7% apoptosis, FI of 85 for MMP, FI of 21 for intracellular free Ca^2+^ and 15 U/mgprot for Ca^2+^-Mg^2+^-ATPase activity. Treatment of the cells with DOX alone resulted in significantly decreased viability (70%), increased apoptosis (21%), decreased MMP (FI of 43), increased intracellular free Ca^2+^ (FI of 30) and decreased Ca^2+^-Mg^2+^-ATPase activity (7 U/mgprot). Compared to the cells exposed to DOX alone, in cells exposed to RF at 12 µW/cm^2^ +DOX, there was a significant increase in viability (83%), decrease in apoptosis (17%), increase in MMP (FI 64), decrease in intracellular free Ca^2+^ (FI of 24) and significant increase in Ca^2+^-Mg^2+^-ATPase activity (11 U/mgprot).

**Table 1:** The viability, apoptosis, mitochondrial membrane potential (MMP), intracellular free Ca^2+^ and Ca^2+^-Mg^2+^-ATPase activity in HL-60 cells.

Group	Viability	Apoptosis	MMP	Intracellular Free Ca^2+^	Ca^2+^-Mg^2+^-ATPase activity
	%	%	(FI*)	(FI)	(U/mgprot)
1. Un-exposed Controls	100±1.5	5.8±1.0	87.6±2.5	18.1±0.9	17.1±0.4
2. Sham-exposed Controls	99.5±2.1	6.2±0.9	87.3±1.9	17.5±1.4	16.7±1.3
3. 900 MHz RF, 12 µW/cm^2^	98.8±3.1	6.7±1.1	85.3±2.0	21.2±0.7	14.5±1.9
4. 0.125 mg/L DOX	70.2±0.2	21.0±1.4	43.0±1.0	30.3±0.2	6.8±0.3
5. 900 MHz RF, 12 µW/cm^2^ + DOX	82.8±2.1	17.1±0.8	63.6±1.4	24.1±3.5	10.9±0.3
6. 900 MHz RF, 120 µW/cm^2^ + DOX	60.7±0.5	-	-	-	-
7. 900 MHz RF, 1200 µW/cm^2^ + DOX	58.6±0.5	-	-	-	-
Group 1 versus 2	*p*>0.05	*p*>0.05	*p*>0.05	*p*>0.05	*p*>0.05
Group 1 versus 3	*p*>0.05	*p*>0.05	*p*>0.05	*p*<0.05	*p*<0.05
Group 2 versus 3	*p*>0.05	*p*>0.05	*p*>0.05	*p*<0.05	*p*<0.05
Group 1 versus 4	*p*<0.01	*p*<0.01	*p*<0.01	*p*<0.01	*p*<0.01
Group 2 versus 4	*p*<0.01	*p*<0.01	*p*<0.01	*p*<0.01	*p*<0.01
Group 3 versus 4	*p*<0.01	*p*<0.01	*p*<0.01	*p*<0.01	*p*<0.01
Group 4 versus 5	*p*<0.01	*p*<0.01	*p*<0.01	*p*<0.01	*p*<0.01

Number of experiments: 2. Data are Mean +/− Standard Deviation.

* FI: Fluorescence Intensity measured in flow cytometer.

## Discussion

We have earlier reported that pre-exposure of mice to 900 MHz at 120 µW/cm^2^ power density for 14 days had a protective effect in hematopoietic tissue following γ-irradiation [10], [11]. The aim of the current study is to determine whether a similar ‘protective’ response would be observed when we utilize promyelocytic leukemic HL-60 cells (derived from hematopoietic tissue) which have been used as a model system for hematopoietic cancers for therapeutic and mechanistic studies [14]. HL-60 cells were pre-exposed to 900 MHz at 12 µW/cm^2^ power density for 3 days and then treated with the chemotherapeutic drug, DOX (0.125 mg/L). Several end-points related to toxicity were examined, viz., viability, apoptosis, MMP, intracellular free Ca^2+^ and Ca^2+^-Mg^2+^-ATPase activity.

Cell populations are under tight control between viability/proliferation and death. Any external/internal influence upon each of these processes may lead to uncontrolled cell growth or cell death [15]. Apoptosis is an evolutionarily conserved and genetically regulated form of cell suicide which plays an important role in the maintenance of tissue homeostasis and in the elimination of mutated or transformed cells from the body. In order to survive, cancer cells and their precursors must develop highly efficient and multiple mechanisms to avoid apoptosis. Thus, the avoidance of apoptosis is regarded as one of the hallmarks of cancer cells [16]. Hence, induction of apoptosis is one of the strategies used in the development of anticancer drugs. Mitochondria are reported to play a major role in apoptosis by opening of the permeability transition pores (PTP). Disruption of MMP affects the electron transport system, oxidative phosphorylation and activation of apoptosis-inducing factors (caspases). A decrease in MMP is generally considered as an early event in many forms of apoptosis [17]–[20]. The observations reported in several studies suggested that increased intracellular calcium (Ca^2+^), as a second messenger, plays an important role in many physiological processes including the triggering of apoptosis in response to a variety of external and internal stimuli. Enhanced intracellular Ca^2+^ concentration might perturb the calcium homeostasis, resulting in cellular calcium overload which is believed to be involved in the apoptosis process. Besides, intracellular Ca^2+^ concentration seems to depend mainly on Ca^2+^ transport system found in plasma membrane, where Ca^2+^-Mg^2+^-ATPase pumps cytosolic calcium to the extra-cellular space and thus maintain homeostasis [21]–[25].

The experimental protocols used in the present study were to examine all of the above interrelated events in HL-60 cells which were pre-exposed to 900 MHz RF at 12 µW/cm^2^ power density and subsequently subjected to a single DOX treatment. The data indicated no significant differences between the cells which were un-exposed, sham-exposed and pre-exposed to RF exposure alone. DOX treatment alone significantly reduced the viability of the cells, induced apoptosis, disrupted MMP, increased intracellular Ca^2+^ concentration by decreasing the Ca^2+^-Mg^2+^-ATPase activity. Thus, the data suggested that apoptosis may be involved in the toxic effects exerted by DOX. In contrast, compared with the cells treated with DOX alone, the response in HL-60 cells which were pre-exposed to RF and then treated with DOX was similar to that in un-treated and sham-exposed controls. Thus, the observations suggested the ability of RF pre-exposure to ‘protect’ the HL-60 cells from the toxicity induced by subsequent DOX treatment.

In RF literature, there were some reports in which researchers have used low SARs and reported biological effects. The following discussion deals with two such investigations. Persson et al [26] have used transverse electromagnetic (TEM) cell to expose rats to 915 MHz RF at 4×10^−4^ to 8×10^−3^ W/kg SAR for 2 minutes to 960 minutes and observed significant leakage in blood-brain-barrier. Nittby et al [27] have also used TEM cell to exposed rats to 900 MHz RF at 0.0006–0.06 W/kg SAR for 2 hours/week for 55 weeks and reported impaired memory. The results obtained in our study can not be compared with the observations in these reports because of the obvious differences in exposure set up, SARs and the parameters used for the assessment of RF exposure. Besides, our study included pre-exposure of HL-60 cells to RF followed by treatment with DOX. Nonetheless, considering a realistic situation, it would be useful to repeat this study at more appropriate SAR level(s).

There were several reports in the literature in which researchers used a sequential exposure of RF and chemotherapeutic drugs, *in vitro* and *in vivo*, to examine the extent of genotoxicity in animal and human cells. The results varied from highly reproducible synergistic effect, very weak synergistic effect, inconsistent effect and no effect [reviewed in 28]. There was one report in which DOX was used to treat human B-cell lymphoblastoid cells immediately before, during or after 1800 MHz RF exposure at 2 W/kg SAR and the extent of single strand breaks in the DNA was determined [29]. The conclusion was that RF exposure alone did not induce DNA damage but, it had an impact on the repair of such damage induced by DOX. Although the experimental protocols used in our study were not similar, RF pre-exposure might have modulated DNA repair processes in HL-60 cells which might have played a role in the decreasing the toxicity induced by DOX. In the context of DNA damage, we have recently reported that mice which were pre-exposed to 900 MHz RF at 120 µW/cm^2^ power density for 4 hours/day for 1, 3, 5, 7 and 14 days and then subjected to gamma-irradiation showed progressively decreased extent of single strand breaks in the DNA of bone marrow leukocytes as compared to those exposed to gamma irradiation alone [30]. The overall results in our current study indicated that RF pre-exposure was able to protect HL-60 cells from the subsequent toxic effects exerted by DOX. Thus, the data suggest the potential of RF to induce adaptive response in HL-60 cells.

## References

[1] (2012) Section of cancer information. International Agency for Cancer Research/WHO. GLOBOCAN 2008 website. Available: http://www.iarc.fr/en/research-groups/sec1/index.php. Accessed: 2 September 2012.

[2] (2012) Cancer facts and 2012. American Cancer Society website. Available: http://www.cancer.org/acs/groups/content/epidemiologysurveilance/documents/document/acspc-031941.pdf. Accessed: 2 September 2012.

[3] Comparisons F (2010) Drug Facts and Comparisons. Lippincott Williams & Wilkins. 3408 p.

[4] Beer MH, Porter RS, Jones TV (2006) The Merck Manual of Diagnosis and Therapy. New Jersey: Merck Research Laboratories, Whitehouse Station. 3000 p.

[5] Carter SK (1975) Adriamycin - a review. J Natl Cancer Inst 55: 1265–1274. 1107570 10.1093/jnci/55.6.1265

[6] Arcamone F (1981) Doxorubicin: Anticancer Antibiotics. New York: Academic Press. 369 p.

[7] Mortazavi S, Mosleh-Shirazi M, Tavassoli A, Taheri M, Bagheri Z, et al (2011) A comparative study on the increased radioresistance to lethal doses of gamma rays after exposure to microwave radiation and oral intake of flaxseed oil. Iran J Radiat Res 9: 9–14.

[8] Sannino A, Sarti M, Reddy SB, Prihoda TJ, Vijayalaxmi, et al (2009) Induction of adaptive response in human blood lymphocytes exposed to radiofrequency radiation. Radiat Res 171: 735–742. 19580480 10.1667/RR1687.1

[9] Sannino A, Zeni O, Sarti M, Romeo S, Reddy SB, et al (2011) Induction of adaptive response in human blood lymphocytes exposed to 900 MHz radiofrequency fields: influence of cell cycle. Int J Radiat Biol 87: 993–999. 21557704 10.3109/09553002.2011.574779

[10] Cao Y, Xu Q, Jin ZD, Zhang J, Lu MX, et al (2010) Effects of 900-MHz microwave radiation on x-ray-induced damage to mouse hematopoietic system. J Toxicol Environ Health A 73: 507–513. 20391130 10.1080/15287390903523451

[11] Cao Y, Xu Q, Jin ZD, Zhou Z, Nie JH, et al (2011) Induction of adaptive response: Pre exposure of mice to 900 MHz radiofrequency fields reduces hematopoietic damage caused by subsequent exposure to ionizing radiation. Int J Radiat Biol 87: 720–728. 21294690 10.3109/09553002.2010.550981

[12] Burkhardt M, Pokovic K, Gnos M, Schmid T, Kuster N (1996) Numerical and experimental dosimetry of Petri dish exposure setups. Bioelectromagnetics 17: 483–493. 8986366 10.1002/(SICI)1521-186X(1996)17:6<483::AID-BEM8>3.0.CO;2-#

[13] Cossarizza A, Baccarani Contri M, Kalashnikova G, Franceschi C (1993) A new method for the cytofluorimetric analysis of mitochondrial membrane potential using the J-aggregate forming lipophilic cation 5,5′,6,6′-tetrachloro-1,1′,3,3′-tetraethylbenzimidazolcarbocyanine iodide (JC-1). Biochem Biophys Res Commun 197: 40–50. 8250945 10.1006/bbrc.1993.2438

[14] Drexler HG, Quentmeier H, MacLeod RAF (2005) Cell line models of leukemia. Drug Discov Today: Disease Models 2: 51–56.

[15] Rezaei PF, Fouladdel S, Cristofanon S, Ghaffari SM, Amin GR, et al (2011) Comparative cellular and molecular analysis of cytotoxicity and apoptosis induction by doxorubicin and Baneh in human breast cancer T47D cells. Cytotechnology 63: 503–512. 21818667 10.1007/s10616-011-9373-6 PMC3176936

[16] Hanahan D, Weinberg RA (2011) Hallmarks of cancer: the next generation. Cell 144: 646–674. 21376230 10.1016/j.cell.2011.02.013

[17] Desagher S, Martinou JC (2000) Mitochondria as the central control point of apoptosis. Trends Cell Biol 10: 369–377. 10932094 10.1016/s0962-8924(00)01803-1

[18] Zamzami N, Marchetti P, Castedo M, Zanin C, Vayssiere JL, et al (1995) Reduction in mitochondrial potential constitutes an early irreversible step of programmed lymphocyte death in vivo. J Exp Med 181: 1661–1672. 7722446 10.1084/jem.181.5.1661 PMC2192017

[19] Kroemer G, Zamzami N, Susin SA (1997) Mitochondrial control of apoptosis. Immunol Today 18: 44–51. 9018974 10.1016/s0167-5699(97)80014-x

[20] Armstrong JS (2006) Mitochondrial membrane permeabilization: the sine qua non for cell death. Bioassays 28: 253–260. 10.1002/bies.20370 16479581

[21] Hajnóczky G, Davies E, Madesh M (2003) Calcium signaling and apoptosis. Biochem Biophys Res Commun 304: 445–454. 12729578 10.1016/s0006-291x(03)00616-8

[22] Xie SQ, Zhang ZQ, Hu GQ, Xu M, Ji BS (2008) HL-37, a novel anthracene derivative, induces Ca^2+^-mediated apoptosis in human breast cancer cells. Toxicology 254: 68–74. 18948164 10.1016/j.tox.2008.09.021

[23] Kluck RM, McDougall CA, Harmon BV, Halliday JW (1994) Calcium chelators induce apoptosis: evidence that raised intracellular ionized calcium is not essential for apoptosis. Biochim Biophys Acta 1223: 247–254. 8086495 10.1016/0167-4889(94)90233-x

[24] Chakraborti S, Das S, Kar P, Ghosh B, Samanta K, et al (2007) Calcium signaling phenomena in heart diseases: a perspective. Mol Cell Biochem 298: 1–40. 17119849 10.1007/s11010-006-9355-8

[25] Delgado-Coello B, Trejo R, Mas-Oliva J (2006) Is there a specific role for the plasma membrane Ca^2+^-ATPase in the hepatocyte? Mol Cell Biochem 285: 1–15. 16477375 10.1007/s11010-005-9060-z

[26] Persson BRR, Salford LG, Brun A (1997) Blood-brain barrier permeability in rats exposed to electromagnetic fields used in wireless communication. Wirel Netw 3: 455–461.

[27] Nittby H, Grafstrom G, Tian DP, Malmgren L, Brun A, et al (2008) Cognitive impairment in rats after long-term exposure to GSM-900 mobile phone radiation. Bioelectromagnetics 29: 219–232. 18044737 10.1002/bem.20386

[28] Verschaeve L, Juutilainen J, Lagroye I, Miyakoshi J, Saunders R, et al (2010) In vitro and in vivo genotoxicity of radiofrequency fields. Mutat Res 705: 252–268. 20955816 10.1016/j.mrrev.2010.10.001

[29] Chen ZJ, Li XX, Lu YZ, Chen SJ, Jian LF, et al (2010) Impact of 1.8-GHz radiofrequency radiation (RFR) on DNA damage and repair induced by doxorubicin in human B-cell lymphoblastoid cells. Mutat Res 695: 16–21. 19833226 10.1016/j.mrgentox.2009.10.001

[30] Jiang B, Nie J, Zhou Z, Zhang J, Tong J, et al (2012) Adaptive response in mice exposed to 900 MHz radiofrequency fields: primary DNA damage. PLoS One 7: e32040. 22389679 10.1371/journal.pone.0032040 PMC3289639

